# Soil, leaf and fruit nutrient data for pear orchards located in the Circum-Bohai Bay and Loess Plateau regions

**DOI:** 10.1038/s41597-023-01999-2

**Published:** 2023-02-11

**Authors:** Mingde Sun, Yanyan Zhao, Zhenxu Liang, Yang Wu, Ruirui Du, Jun Liu, Futong Yu, Songzhong Liu

**Affiliations:** 1 Institute of Forestry & Pomology, Beijing Academy of Agriculture & Forestry Sciences, Beijing Engineering Research Center for Deciduous Fruit Trees, Key Laboratory of Biology and Genetic Improvement of Horticultural Crops (North China), Ministry of Agriculture, Beijing, 100093 China; 2 College of Resources and Environmental Sciences, China Agricultural University, Beijing, 100193 China

**Keywords:** Plant physiology, Agroecology

## Abstract

The data described in this paper were collected from the Circum-Bohai Bay and Loess Plateau regions of northern China. Soil, leaf and fruit nutrients from 225 typical pear orchards in these regions were measured. Soil data included pH, organic matter, total N, alkaline hydrolysable N, available P and available K concentrations of 3 different soil layers, 0–20 cm, 20–40 cm and 40–60 cm, from different orchards. Leaf and fruit data included N, P, K, Ca, Fe, Mn, Cu, Zn and B concentrations of pear trees from different orchards. These data can be used to assess the soil nutrient supply and leaf and fruit nutrient status of pear orchards in two major producing areas, Circum-Bohai Bay and Loess Plateau. Additionally, this dataset provides data to support the development of regionalized and standardized soil nutrient management programs for pear orchards, as well as regionalized layouts of the main varieties in the two producing areas.

## Background & Summary

China is the largest pear producer in the world. The main areas in which pear trees are distributed in China are the Circum-Bohai Bay, Loess Plateau and Yangtze River Basin regions, among which Circum-Bohai Bay and Loess Plateau are located in northern China. The cultivated area and yield of pears planted in these two regions are 338 kha and 7.96 Gkg, accounting for 24.53% and 33.29% of the world’s total cultivated area and total yield, respectively^1^.

The Circum-Bohai Bay pear producing area includes the Hebei, Shandong, and Liaoning provinces and Beijing. This area contains various landforms, such as plains, hills and piedmont slopes, with various soil types, such as brown soil, cinnamon soil and fluvo-aquic soil. The pear varieties in these regions are diverse, including Yali (*Pyrus bretschneideri* Rehd.) and Xuehuali (*Pyrus bretschneideri* Rehd.) in Hebei, Laiyangchili (*Pyrus bretschneideri* Rehd.) and *Pyrus communis* L. in Beijing and Nanguoli (*Pyrus ussuriensis* Maxim.) in Liaoning. The Loess Plateau pear producing area is located on Loess Plateau. It includes all of Shanxi and the Shaanxi Provinces and parts of Gansu Province and is approximately 1000 m above sea level, with sufficient light, a large temperature difference between day and night, a deep loess layer, large terrain variations, frequent droughts and a lack of irrigation. Dangshansuli (*Pyrus bretschneideri* Rehd.) and Yuluxiangli (*Pyrus bretschneideri* Rehd.) are the main cultivated varieties in this region.

Fruit mineral nutrient concentrations are not only closely related to fruit quality, nutritional value and physiological diseases during storage^2,3^, but also play a key role in promoting human health^4^. Pear tree growth and fruit yield and quality are greatly affected by soil conditions and tree nutrient status, which can be reflected in the leaf mineral nutrient status^5^ and fruit nutrient status^6^. Researchers have investigated and studied the soil nutrient status^7^, the leaf nutrient status^8^ and the relationship between soil nutrients and leaf nutrients^9^ of pear orchards in parts of Circum-Bohai Bay and Loess Plateau, which play an important role in guiding pear production. However, the relevant datasets on fruit nutrition of major cultivars planted in Circum-Bohai Bay and Loess Plateau regions are missing and the datasets on leaf and soil nutrients are incomplete. Thus, we present datasets of soil available nutrient concentrations and leaf and fruit mineral nutrient concentrations of 225 typical pear orchards in Circum-Bohai Bay and Loess Plateau to provide guidance for the accurate management of pear tree nutrients in these regions.

This dataset can be used to obtain a more in-depth and systematic understanding of the soil nutrient supply and leaf and fruit nutrient status of pear trees in the two major producing areas. This dataset can be analysed in a variety of ways, such as by correlation analysis of leaf mineral nutrient concentration and soil mineral nutrient concentration, and by multivariate statistical analysis and partial least squares regression analysis on fruit mineral nutrient concentration and soil mineral nutrient concentration. These analyses will be helpful in clarifying the influence of the main mineral soil nutrient factors on leaf and fruit mineral concentrations in Circum-Bohai Bay and Loess Plateau regions and then determine the optimum soil mineral nutrient concentration that ensures abundant mineral concentrations in leaves and fruits for high quality and high yield. In addition, prominent problems, such as large differences in nutrient management and slow improvement in fruit yield and quality caused by natural factors such as topography, soil types, irrigation conditions and main varieties, can be eliminated by comparing the differences in soil mineral nutrient management between the Circum-Bohai Bay and Loess Plateau regions. This study provides theoretical support for the development of regionalized and standardized soil nutrient management programs in pear orchards, as well as for a regionalized layout of the main varieties in the two producing areas.

## Methods

### Orchard site selection

**Fig. 1:**
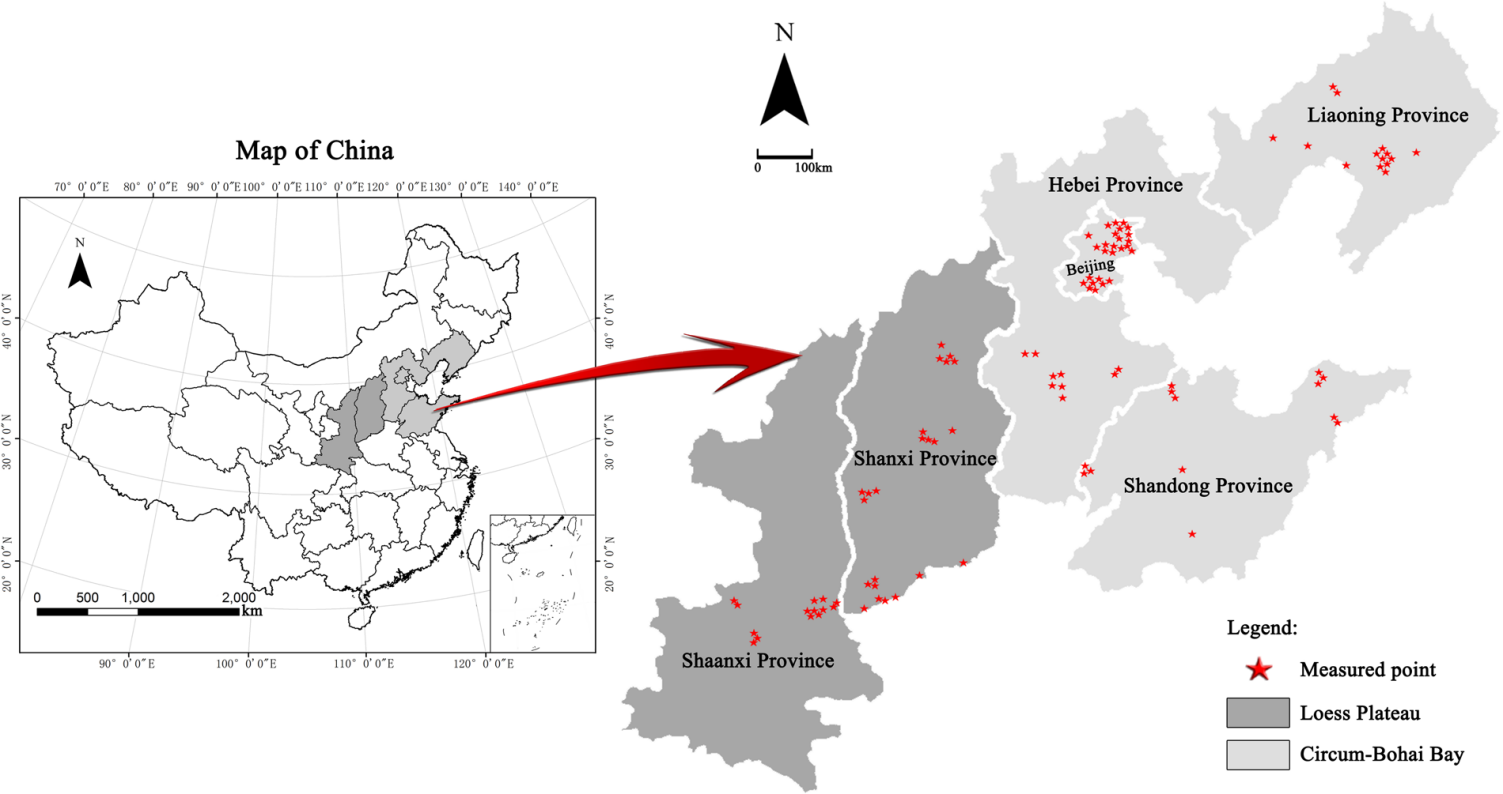
The locations of the 225 pear orchards.

The survey was conducted from 2018 to 2019 in the Circum-Bohai Bay region, which included Shandong, Hebei, and Liaoning provinces and Beijing, and the Loess Plateau region, which included Shanxi and Shaanxi provinces. Five typical production counties were selected in each province or city. Representative orchards were selected according to the production of the main varieties in each county (orchard area was greater than 1.0 ha; the pear trees were 15 to 25 years old; and the yield of orchards ranged from 40 to 60 t ha^−1^). A total of 225 orchards were investigated (Fig. 1), including 150 in the Circum-Bohai Bay region and 75 in the Loess Plateau region (Table 1).

**Table 1:** Numbers of pear orchard and main cultivated varieties investigated in Circum-Bohai Bay and Loess Plateau.

Investigated region		Number of orchards	Main cultivated pear varieties
Circum-Bohai Bay	Shandong	39	Yali (*Pyrus bretschneideri* Rehd.) (18)
			Laiyangchili (*Pyrus bretschneideri* Rehd.) (8)
			Dr. Jules. Guyot (*Pyrus communis* Linn.) (8)
			Whangkeumbae (*Pyrus pyrifolia* Nakai.) (5)
	Hebei	47	Yali (*Pyrus bretschneideri* Rehd.) (47)
	Beijing	42	Yali (*Pyrus bretschneideri* Rehd.) (16)
			Whangkeumbae (*Pyrus pyrifolia* Nakai.) (26)
	Liaoning	22	Nanguoli (*Pyrus ussuriensis* Maxim.) (22)
Loess Plateau	Shanxi	44	Dangshansuli (*Pyrus bretschneideri* Rehd.) (32)
			Yuluxiangli (*Pyrus bretschneideri* Rehd.) (12)
	Shaanxi	31	Dangshansuli (*Pyrus bretschneideri* Rehd.) (31)

The numbers in parentheses represent the number of orchards.

### Sample collection and pretreatment

**Fig. 2:**
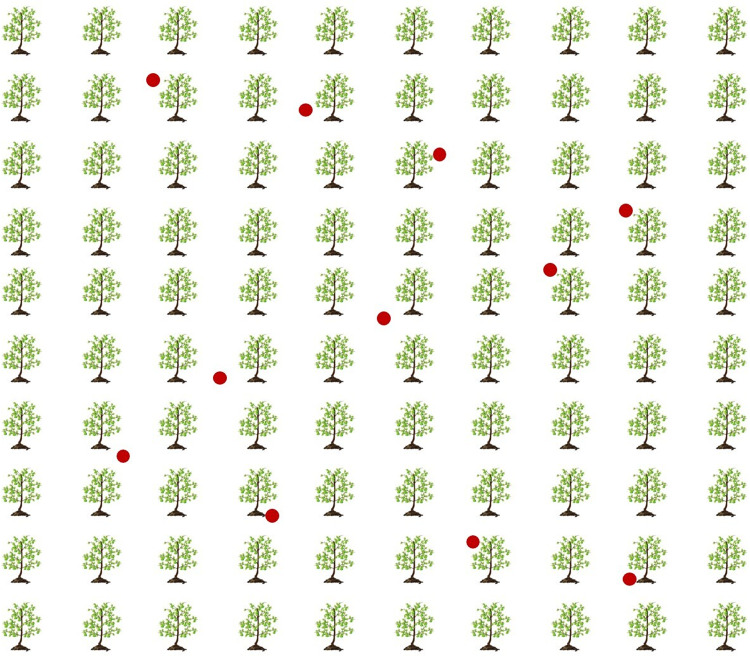
The “S” shape sampling method. The red dots are the sampling locations.

Soil and leaf samples were collected at the stage in which the growth of new shoots ceased, from July 1 to July 15^10^. Eleven sampling sites were determined in each orchard according to an “S” shape sampling method (Fig. 2), and soil samples from the 0–20 cm, 20–40 cm and 40–60 cm layers were collected. The soil samples of the same soil layer at each sampling site were mixed into one sample. Then, the soil samples were air-dried, ground and sifted with a nylon sieve for determination of nutrient concentrations.

Ten to fifteen pear trees in each orchard of the same size and vigour and 5 to 10 mature leaves from the middle of a long shoot from the periphery of each tree were selected for leaf sampling^11^. Then, all the leaves from the same orchard were mixed into one leaf sample. The leaves were washed with tap water containing a detergent, with deionized water, with 0.01 M hydrochloric acid and then with deionized water again and then dried at 100 °C for 30 min and at 70 °C to a constant weight. Then, the leaf samples were crushed into a powder and sifted with a nylon sieve for nutrient determination.

Fruit samples were collected at the ripening stage. Pear trees from which leaf samples were collected from each orchard were selected for fruit sample collection. Three to five peripheral fruits of the same size were collected from each tree, and fruit samples from the same orchard were mixed into one sample. The fruits were washed with tap water containing a detergent, with deionized water, with 0.01 M hydrochloric acid and then with deionized water again, cut into slices and then dried at 100 °C for 30 min and at 70 °C to a constant weight. Then, the fruit samples were crushed into a powder and sifted with a nylon sieve for nutrient determination.

### Sample determination

Various indicators of soil and plant samples were determined according to the method of Cui *et al*.^12^ and Bao^13^.

#### Soil pH determination

A potentiometric method was used to measure soil pH. Carbon dioxide-free water was added to soil that had been passed through a 2 mm sieve at a water-soil ratio of 2.5:1. The soil solution was stirred for 1 min and left undisturbed for 30 min. Each soil sample was measured more than three times with a pH meter (FE20K PLUS PH, Mettler-Toledo, Switzerland), and the difference in the parallel determination results was less than 0.2 pH units. The electrode was washed with deionized water and dried with filter paper after each sample measurement. A calibration solution was used to calibrate the electrode between measurements after every 10 soil samples.

#### Soil organic matter determination

Soil organic matter was measured according to the Schollenberger method using chromic acid redox titration. Five millilitres of a 0.8 M 1/6 K_2_Cr_2_O_7_ solution was added to a test tube with approximately 0.5000 g of soil that had been passed through a 0.25 mm sieve. The mixture was then added to 5 mL concentrated sulfuric acid and shaken gently to disperse the soil. The tube was placed in a phosphoric acid bath, heated to 170 °C and boiled for 5 min. To condense the water vapour that escaped during the heating process, a small funnel was placed on the top of the test tube. The substances in the test tube and funnel were transferred to a conical flask after cooling. Then, the solution was added to 1,10-phenanthroline hydrate and titrated with 0.2 M FeSO_4_ until it turned maroon. A blank experiment was performed when each batch of samples was measured. The soil organic matter content was calculated according to the following formula:

1 $${\rm{\omega }}\left({\rm{OM}}\right)=\frac{\left({\rm{V}}-{\rm{V}}0\right)\times {\rm{c}}\times 3\times 1.724\times {\rm{f}}}{{\rm{m}}}$$

ω(OM): soil organic matter content; c: standard FeSO_4_ solution concentration; V: volume of the standard FeSO_4_ used in titration; V0: volume of standard FeSO_4_ used in titrating control sample; 3: molar mass of a quarter of carbon; 1.724: the conversion factor from organic carbon to organic matter; f: oxidation correction coefficient (the value was 1.1); m: mass of oven-dried soil sample.

#### Soil total N determination

Total N was determined by the semitrace Kjeldahl method. Approximately 1.0000 g of air-dried soil that had been passed through a 0.25 mm sieve was added to a digestion tube. Meanwhile, the soil moisture content was measured to calculate the mass of the oven-dried soil. Two grams of accelerator and 5 mL of concentrated sulfuric acid were added to the tube. The tube was then covered with a small funnel, and the sample was digested at 360 °C for 15–20 min. The mixture was digested for 1 h until the colour changed from brown to greyish green or greyish white. Two digested soilless samples were used as controls. After the digestion tube cooled, it was placed in a distiller, and a small amount of deionized water was added. Five millilitres of a 2% boric acid indicator was added to a 150 mL conical flask, and the flask was placed at the end of the condenser tube. Then, the digestion solution was distilled until the distillate volume was approximately 75 mL. The distillate was titrated with 0.01 M standard hydrochloric acid to a purplish red colour endpoint. The soil total N concentration was calculated according to the following formula:

2 $${\rm{\omega }}({\rm{N}})=\frac{({\rm{V}}-{\rm{V}}0)\times {\rm{c}}\times 14}{{\rm{m}}}$$

ω(N): soil total N concentration; c: standard acid concentration; V: volume of the standard acid used in titration; V0: volume of standard acid used in titrating control sample; 14: molar mass of N; m: mass of oven-dried soil sample.

#### Soil alkaline hydrolysable N determination

Approximately 2.00 g of air-dried soil that have been passed through a 2 mm sieve was placed in the outer chamber of a diffuser. The diffuser was gently rotated to evenly distribute the soil in the outer chamber. Two millilitres of H_3_BO_3_ indicator was placed in the inner chamber of the diffusion dish. The edge of the frosted glass surface of the diffuser was coated with alkaline glycerin and covered with frosted glass. The diffuser was covered tightly and secured with rubber bands after 10.00 mL of 1 M NaOH was injected into the diffuser through a hole in the frosted glass. The diffuser was placed in a 40 °C incubator for alkaline hydrolysis diffusion for 24 h. Then, the mixture was titrated with 0.01 M standard hydrochloric acid until it turned purplish red. A blank test was performed at the same time as the samples. The soil alkaline hydrolysable N concentration was calculated according to the following formula:

3 $${\rm{\omega }}({\rm{N}})=\frac{({\rm{V}}-{\rm{V}}0)\times {\rm{c}}\times 14}{{\rm{m}}}$$

ω(N): soil alkaline hydrolysable N concentration; c: standard acid solution concentration; V: volume of the standard acid used in titration; V0: volume of standard acid used in titrating control sample; 14: molar mass of N; m: mass of air-dried soil sample.

#### Soil available P determination

Approximately 2.50 g of air-dried soil that had been passed through a 2 mm sieve was placed in a plastic bottle and 50 mL of 0.5 M NaHCO_3_ was added. After the bottle was shaken for 30 min, the mixture was immediately filtered with phosphorus-free filter paper. Ten millilitres of the filtrate was accurately measured into a conical flask, and 5.00 mL of Mo-Sb-Vc colour developer and 10 mL of deionized water were added. The absorbance of the mixture was measured at approximately 700 nm after 30 min using a UV-Vis spectrophotometer (UV1900PC, AuCy Instrument, Shanghai, China). Finally, the P concentration was calculated according to a standard curve prepared with solutions of different P concentrations. A blank test was performed at the same time that the samples were determined.

#### Soil available K determination

Approximately 5.00 g of air-dried soil that had been passed through a 2 mm sieve was placed in a plastic bottle, and 50 mL of 1.0 M NH_4_OAc was added. After the sample was shaken for 30 min, the mixture was immediately filtered with dry filter paper. The concentration of K in the filtrate was determined directly by a flame photometer (LM12-FP6430, Haifuda, China) according to a standard curve prepared with solutions of different K concentrations. A blank test was performed at the same time that the samples were determined.

#### Leaf and fruit N determination

Approximately 0.3000 g of plant powder that had been passed through a 0.5 mm sieve was placed into a digestion tube and 5 mL concentrated sulfuric acid was added. Then, the digestion tube was placed onto a digestion stove at 360 °C after two doses of 2 mL H_2_O_2_, and the sample was digested until the mixture turned brown. After the tube cooled, 2 mL H_2_O_2_ was added, and the digestion was continued for 5 min. This process was repeated until the mixture turned clear. The mixture was diluted to 100 mL in a volumetric flask for testing after it cooled. Then, 5 to 10 mL of the liquid to be tested was accurately measured into a distiller for distillation. The distillation and titration processes were the same as those used for ammonium in the Soil total N determination section. A blank test was performed at the same time as sample measurement. The leaf or fruit N concentration was calculated according to the following formula:

4 $${\rm{\omega }}({\rm{N}})=\frac{({\rm{V}}-{\rm{V}}0)\times {\rm{c}}\times 14\times {\rm{V}}1}{{\rm{m}}\times {\rm{V}}2}$$

ω(N): total N concentration; c: standard acid concentration; V: volume of the standard acid used in titration; V0: volume of standard acid used in titrating control sample; 14: molar mass of N; m: mass of oven-dried sample; V1: volume of the digestion solution after constant volume; V2: measured volume of digestion solution after constant volume.

#### Leaf and fruit P, K, Ca, Fe, Mn, Cu, Zn, B determination

Approximately 0.5000 g of plant powder that had been passed through a 0.5 mm sieve was placed in a digestion tube and a 10 mL mixture of concentrated nitric acid and hypochlorous acid (4:1) was added. After the sample was left undisturbed for more than 4 h, it was placed onto a digestion stove and heated to 150 °C so that NO_2_ could volatilize slowly. Then, the temperature was appropriately increased to a temperature not higher than 250 °C until the digestive solution was transparent and approximately 2 mL remained. The solution was transferred into a volumetric flask after cooling and adjusted to a constant volume of 50 mL. The solution was then filtered, and the concentration of each element in the solution was determined by a plasma emission spectrometer (ICP-OES, OPTIMA 3300 DV, 75 Perkin-Elmer, USA). A blank test was performed at the same time as sample measurement. The leaf or fruit P, K, Ca, Fe, Mn, Cu, Zn, and B concentrations were calculated according to the following formula:

5 $${\rm{\omega }}({\rm{P}},{\rm{K}},{\rm{Ca}},{\rm{Fe}},{\rm{Mn}},{\rm{Cu}},{\rm{Zn}},{\rm{B}})=\frac{\rho ({\rm{P}},{\rm{K}},{\rm{Ca}},{\rm{Fe}},{\rm{Mn}},{\rm{Cu}},{\rm{Zn}},{\rm{B}})\times {\rm{V}}\times {\rm{f}}}{{\rm{m}}}$$

ω(P, K, Ca, Fe, Mn, Cu, Zn, B): P, K, Ca, Fe, Mn, Cu, Zn, B concentration in leaf or fruit; ρ(P, K, Ca, Fe, Mn, Cu, Zn, B): the concentration of P, K, Ca, Fe, Mn, Cu, Zn or B in the liquid to be measured; V: volume of the liquid to be measured after constant volume; f: dilution ratio of the liquid to be measured; m: mass of oven-dried sample.

## Data Records

The dataset is accessible from figshare^14^. The dataset for the soil, leaf and fruit nutrient concentrations of pears in different orchards are displayed in the database. Soil data included pH, organic matter, total N, alkaline hydrolysable N, available P and available K concentrations of 3 different soil layers, 0–20 cm, 20–40 cm and 40–60 cm (named Soil 0–20 cm, Soil 20–40 cm and Soil 40–60 cm, respectively). Leaf and fruit data (named Leaf and Fruit, respectively) included N, P, K, Ca, Fe, Mn, Cu, Zn and B concentrations. In the dataset, CBB represents Circum-Bohai Bay, LP represents Loess Plateau, BJ represents Beijing, HB represents Hebei, SD represents Shandong, LN represents Liaoning, SX represents Shanxi, SaX represents Shaanxi, and different numbers in the same province indicate orchards at different locations.

## Technical Validation

### The investigated area

The Circum-Bohai Bay and Loess Plateau regions are two of the three recognized major pear-producing areas in China. The typical survey counties were collaboratively determined with the local fruit tree scientific research institute or technology extension departments based on comprehensive consideration of the main cultivars and distributions in the provinces, as well as the cultivation area and yield of each county.

### The investigated pear orchards

To ensure the representativeness of the selected orchards, the research team conducted a field investigation and comprehensively considered the areas and yields of the orchards and the growth status and ages of the pear trees in the orchards.

### Investigation method

This survey was carried out by researchers with backgrounds in fruit tree, plant nutrition and soil science. Before sample collection, the investigation team developed a detailed manual for soil, leaf and fruit sample collection based on the biological habits of pear trees. For sample collection, pear trees with essentially the same size and growth states were selected.

### Determination of the samples

The soil samples were pretreated and analysed at the Key Laboratory of Plant-Soil Interaction, Ministry of Education, College of Resources and Environmental Sciences, China Agricultural University. Leaf and fruit samples were pretreated and tested at the Test Center of Vegetable Seed Quality, Ministry of Agriculture. These institutions are authoritative testing agencies. The sample pretreatment and determination methods were in line with testing standards to ensure the accuracy of the sample analysis results.

## Data Availability

No custom code was used in this study.
